# Nitrogen Sources Reprogram Carbon and Nitrogen Metabolism to Promote Andrographolide Biosynthesis in *Andrographis paniculata* (Burm.f.) Nees Seedlings

**DOI:** 10.3390/ijms25073990

**Published:** 2024-04-03

**Authors:** Shaofen Jian, Si Wan, Yang Lin, Chu Zhong

**Affiliations:** 1 National Center for TCM Inheritance and Innovation, Guangxi Botanical Garden of Medicinal Plants, Nanning 530023, China; jsfzc2011@126.com (S.J.); 18078187624@163.com (S.W.); linyangnn@163.com (Y.L.); 2 Guangxi Key Laboratory of Medicinal Resource Protection and Genetic Improvement, Guangxi Botanical Garden of Medicinal Plants, Nanning 530023, China; 3 Guangxi Engineering Research Center of TCM Resource Intelligent Creation, Guangxi Botanical Garden of Medicinal Plants, Nanning 530023, China

**Keywords:** nitrogen source, andrographolide biosynthesis, carbon and nitrogen metabolism, arginine metabolism, phytohormones

## Abstract

Carbon (C) and nitrogen (N) metabolisms participate in N source-regulated secondary metabolism in medicinal plants, but the specific mechanisms involved remain to be investigated. By using nitrate (NN), ammonium (AN), urea (UN), and glycine (GN), respectively, as sole N sources, we found that N sources remarkably affected the contents of diterpenoid lactone components along with C and N metabolisms reprograming in *Andrographis paniculata*, as compared to NN, the other three N sources raised the levels of 14-deoxyandrographolide, andrographolide, dehydroandrographolide (except UN), and neoandrographolide (except AN) with a prominent accumulation of farnesyl pyrophosphate (FPP). These N sources also raised the photosynthetic rate and the levels of fructose and/or sucrose but reduced the activities of phosphofructokinase (PFK), glyceraldehyde-3-phosphate dehydrogenase (GAPDH), phospho*enol*pyruvate carboxylase (PEPC) and pyruvate dehydrogenase (PDH). Conversely, phospho*enol*pyruvate carboxykinase (PEPCK) and malate enzyme (ME) activities were upregulated. Simultaneously, citrate, cis-aconitate and isocitrate levels declined, and N assimilation was inhibited. These results indicated that AN, UN and GN reduced the metabolic flow of carbohydrates from glycolysis into the TCA cycle and downstream N assimilation. Furthermore, they enhanced arginine and GABA metabolism, which increased C replenishment of the TCA cycle, and increased ethylene and salicylic acid (SA) levels. Thus, we proposed that the N sources reprogrammed C and N metabolism, attenuating the competition of N assimilation for C, and promoting the synthesis and accumulation of andrographolide through plant hormone signaling. To obtain a higher production of andrographolide in *A. paniculata*, AN fertilizer is recommended in its N management.

## 1. Introduction

Secondary metabolites represent important defensive substances in plants and the majority source of natural bioactive ingredients in medicinal plants. Plant secondary metabolism is strongly affected by genetic and environmental factors [1], among which nitrogen (N) is a crucial nutrient [2,3] as it constitutes myriads of life macromolecules that play key roles in plant development and metabolism processes. Plants obtain N mainly from soils with the forms of nitrate (NO_3_^−^) and ammonium (NH_4_^+^) [4]. In addition, soils are rich in organic N, such as amino acids and peptides, which also potentially significantly contribute to plant nutrition [5].

N sources strongly affect the accumulation and component of secondary metabolites in medicinal plants. For example, nitrate elevated the synthesis of berberine, jatrorrhizine and palmatine in *Phellodendron amurense* [6], stevioside glycoside in *Stevia rebaudiana* [7], polyphenols in *Urtica dioica* [8], and total flavone, volatile oil and rosmarinic acid in *Perilla frutescens* [9]. Studies also revealed that ammonium stimulated the accumulation of andrographolide in *Andrographis paniculata* [10] and phenolic metabolites in *Matricaria chamomilla* [11]. Apparently, the preferable N sources required for the accumulation of secondary metabolites varied greatly depending on plant species and chemicals.

Secondary metabolites (including N-containing organic compounds, terpenoids, and phenolic compounds) are derived from primary metabolism pathways such as tricarboxylic acid (TCA) cycle, methylerythritol phosphate pathway (MEP), and mevalonic and shikimic acid pathways [12], among which the TCA cycle bridges the C and N metabolisms and therefore is in the center of regulation on primary and secondary metabolism. The mechanisms by which N sources regulate secondary metabolism in medicinal plants at least include (1) modulating the expression of genes in the synthetic pathway of secondary metabolites [13] and (2) reprogramming primary C and N metabolism. The uptake and assimilation of different N sources affect cytoplasmic pH [14]. Assimilation of NH_4_^+^ results in the acidification of cytoplasm [15]. It has been proposed that cytoplasmic pH is the primary factor inducing secondary metabolism in plants [16]. The C-N status regulates plant secondary metabolism [17]. N sources modulate plant metabolite profiles by influencing the expression and activity of enzymes in C and N metabolisms [10,18,19]. The enzymes PEPC, ICDH, MDH, and GDH were involved in NH_4_^+^ trigged alteration of C and N metabolisms [20]. Generally, the metabolic flow that tends to increase carbohydrate accumulate and decrease protein synthesis elevates the C/N ratio in plant tissues [21], which is positively correlated with the concentration of secondary metabolites [21,22,23]. It has also been widely corroborated that higher accumulation and exogenous application of sugars promote secondary metabolite biosynthesis in medicinal plants [7,10,21,24,25,26]. To date, however, deciphering the physiological and molecular rationale of N sources on the secondary metabolism of medicinal plants is still challenging due to the complexity of metabolic regulation in plants.

*Andrographis paniculata* is an important international traditional medicinal plant that originated from South Asia and is widely cultivated in Southeast Asia and South China. It is an herb traditionally used to treat cold, fever, and laryngitis [27]. Its major bioactive ingredients, andrographolide and its derivatives are potential drugs for anticancer, antibacterial, anti-hepatitis and antivirus [28,29], including COVID-19 [30,31,32]. Although the andrographolide biosynthetic pathway has been partially worked out [33,34], only a small amount of information is available on its regulatory mechanism, especially in the aspect of cultivation, including N regimes.

Combining metabolomics and physiological and transcriptional analyses, we aimed to reveal the physiological and molecular mechanism by which N sources triggered metabolic reprogramming in contribution to andrographolide biosynthesis in *A. paniculata*. We found that N sources affected andrographolide biosynthesis by manipulating C metabolic fluxes from glycolysis into the TCA cycle and downstream N assimilation. The findings provide new insight into the intricate relationship between N sources, C metabolism, and secondary metabolism in medicinal plants.

## 2. Results

### 2.1. Diterpenoid Lactone Compounds Affected by N Sources

**Figure 1:**
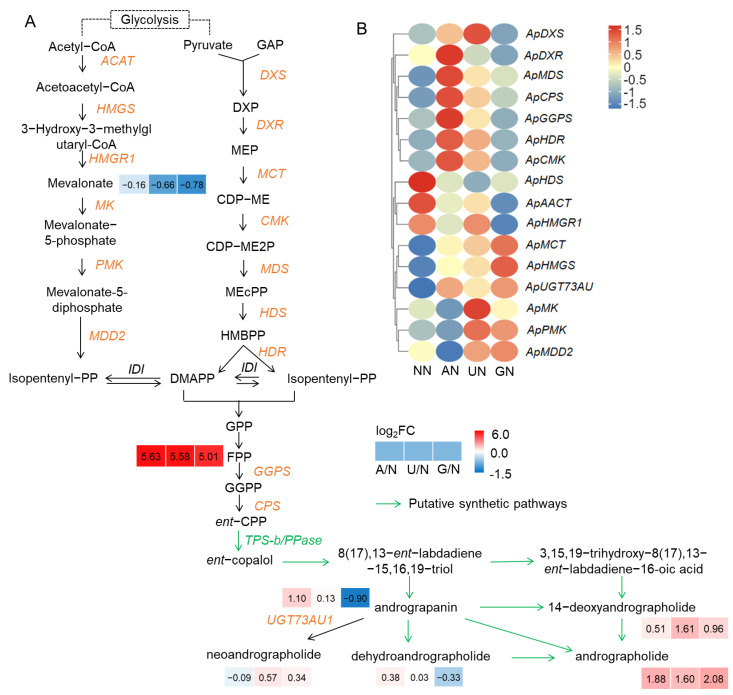
Analysis of the biosynthetic pathway of active diterpene lactones in *A. paniculata*. (**A**) The biosynthetic pathway of active diterpene lactones in *A. paniculata*. Confirmed genes in the synthetic pathways were labeled brown and italic. Green arrows indicate speculative synthetic pathways. Data in boxes indicate log_2_FC values of AN, UN, and GN in relation to NN, respectively (*n* = 3). (**B**) Heatmap of the expression of genes in the synthetic pathways of andrographolide components. The data were normalized. NN—nitrate nitrogen; AN—ammonium nitrogen; UN—urea nitrogen; GN—glycine nitrogen.

N sources considerably affected the contents of diterpenoid lactone compounds in *A. paniculata* plants. In comparison to NN, the other three N sources increased 14-deoxyandrographolide and andrographolide levels. In contrast, GN decreased andrograpanin and dehydroandrographolide levels, and AN slightly decreased neoandrographolide levels (Figure 1A). Farnesyl pyrophosphate (FPP) profoundly accumulated, but mevalonate was reduced under AN and organic N conditions.

Different N sources affected the expression of genes in andrographolide biosynthetic pathways differentially. Compared to NN, AN mainly upregulated the expression of genes in the 2-C-methyl-D-erythritol 4-phosphate (MEP) pathway, including *ApDXS*, *ApDXR*, *ApCMK*, *ApMDS*, *ApHDR*, as well as *ApGGPS*, *ApCPS* and *ApUGT73AU*; while the expression of genes in the mevalonate (MVA) pathway were mainly down-regulated. UN increased the expression of genes in MEP and MVA pathways, but the upregulation of *ApGGPS*, *ApCPS* and *ApUGT73AU* was weaker than in AN (Figure 1B). *ApMCT*, *ApHMGS*, *ApPMK*, *ApMDD2*, and *ApUGT73AU1* were upregulated, but other genes were not affected or downregulated by GN (Figure 1B).

### 2.2. Glycolysis and the TCA Cycle

As shown in Figure S1A, the CO_2_ response curve of photosynthesis was slightly different among N sources. *V*_cmax_, *J*_max_ and *TPU* were relatively higher in AN and UN, with significant differences in *V*_cmax_ and *TPU* between AN and GN (Table S2). AN and UN considerably increased the photosynthesis rate of plants at ambient CO_2_ concentration (400 μmol·mol^−1^) (Figure S1B). The chlorophyll content was higher in the plants grown in UN, which was remarkably greater than that in GN (Figure S1C). The specific leaf area (SLA) was remarkably lower in NN than in UN and GN (Figure S1D), while special leaf N (SLN) and photosynthetic N use efficiency (PNUE) were not different among N sources (Figure S1E,F). These results suggested that the photosynthetic productivity of plants grown in NN is relatively lower than that of other N forms.

**Figure 2:**
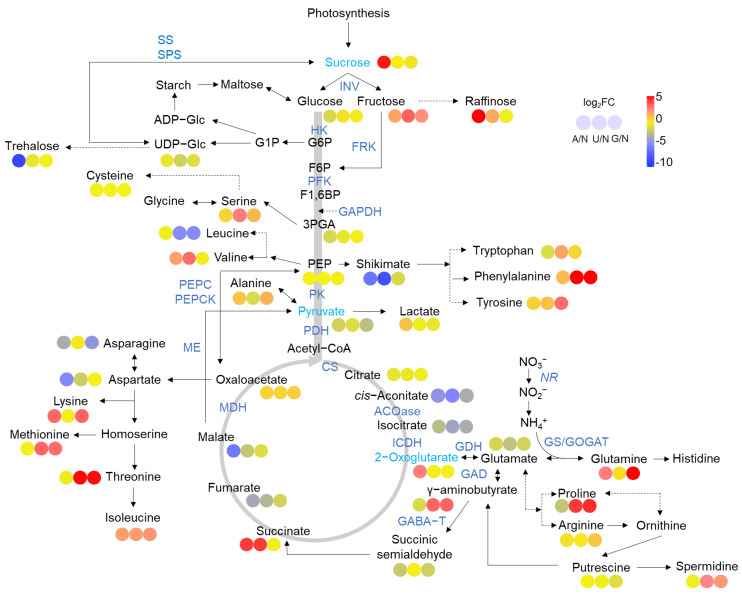
Overview of C and N metabolisms affected by N sources. Heatmaps show the values of log_2_FC. NN was used as a control. Enzymes in the pathways were labeled blue. NN—nitrate nitrogen; AN—ammonium nitrogen; UN—urea nitrogen; GN—glycine nitrogen.

Compared to NN, AN and organic N increased sucrose (AN only) and fructose (Figure 2). In contrast, UDPG and trehalose levels were decreased by AN and organic N. Intermediates of glycolysis such as 3-phosphoglycerate (3-PGA), PEP, and pyruvate were decreased or unchanged by AN and organic N.

**Figure 3:**
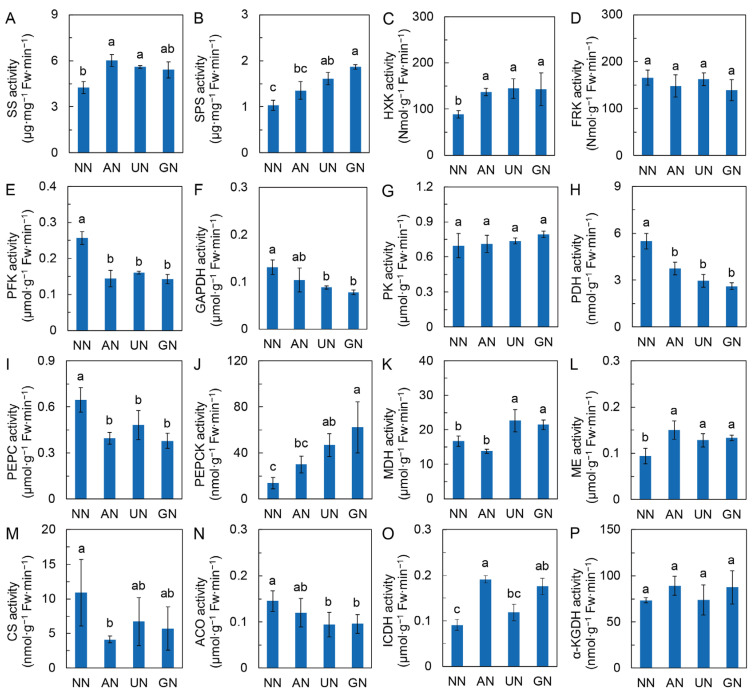
Activities of enzymes in C metabolism. (**A**) Sucrose synthase activity; (**B**) Sucrose phosphate synthase activity; (**C**) Hexokinase activity; (**D**) Fructokinase activity; (**E**) Phosphofructokinase activity; (**F**) Glyceraldehyde-3-phosphate dehydrogenase activity; (**G**) Pyruvate kinase activity; (**H**) Pyruvate dehydrogenase activity; (**I**) PEP carboxylase activity; (**J**) PEP carboxykinase activity; (**K**) Malate dehydrogenase activity; (**L**) Malate enzyme activity; (**M**) Citrate synthase activity; (**N**) Aconitase activity; (**O**) Isocitrate dehydrogenase activity; (**P**) α-ketoglutarate dehydrogenase activity. Data were represented by means ± SD (*n* = 3 or 4). Different letters on bars indicate significant differences at *p* < 0.05 level according to one-way ANOVA combined with Duncan’s multiple range method. NN—nitrate nitrogen; AN—ammonium nitrogen; UN—urea nitrogen; GN—glycine nitrogen.

In comparison to NN, the activity of SS was significantly increased by AN and UN, and SPS activity was significantly increased by UN and GN (Figure 3A,B). The HXK activity was remarkably increased by AN and organic N, while the FRK activity was not different among N sources (Figure 3C,D). However, the activities of PFK and GAPDH were remarkably decreased by AN and/or organic N (Figure 3E,F). The activity of PK was not different among N sources (Figure 3G), while the activities of PDH and PEPC were reduced by AN and organic N (Figure 3H,I). In contrast, the PEPCK activity was remarkably increased by AN and organic N (Figure 3J).

The levels of organic acids in the TCA cycle, including citrate, *cis*-aconitate, isocitrate, fumarate and malate, were reduced. Still, the levels of oxaloacetate, 2-oxoglutarate and succinate were mainly increased by AN. Organic N. Coherently, the activity of MDH was significantly increased in UN and GN, and the ME activity was remarkably increased in AN and organic N (Figure 3K,L). However, the activities of CS and ACO were considerably inhibited by AN and organic N, respectively (Figure 3M,N). The ICDH activity was increased in AN and GN (Figure 3O), while the α-KGDH activity was not significantly different among N sources (Figure 3P). These results suggested that AN and organic N diminished the partitioning of C metabolic flow from glycolysis into the TCA cycle.

### 2.3. N Assimilation and Amino Acids Metabolism

**Figure 4:**
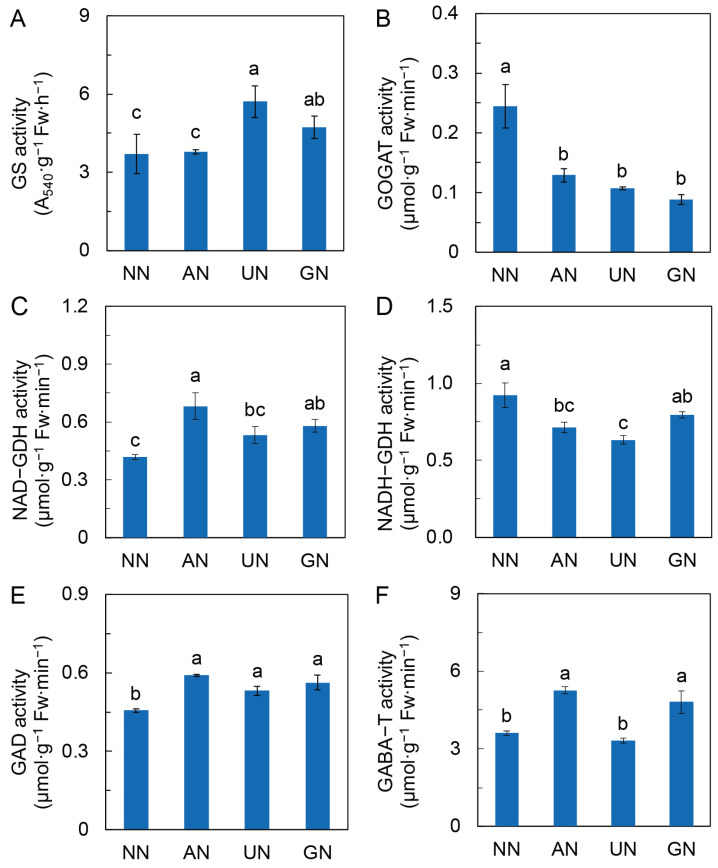
Activities of enzymes in N metabolism. (**A**) Glutamine synthase activity; (**B**) Glutamine oxoglutarate aminotransferase activity; (**C**) NAD-dependent glutamate dehydrogenase activity; (**D**) NADH-dependent glutamate dehydrogenase activity; (**E**) Glutamate decarboxylase activity; (**F**) GABA aminotransferase activity. Data were represented by means ± SD (*n* = 3 or 4). Different letters on bars indicate significant differences at *p* < 0.05 level according to one-way ANOVA combined with Duncan’s multiple range method. NN—nitrate nitrogen; AN—ammonium nitrogen; UN—urea nitrogen; GN—glycine nitrogen.

Glutamine synthase (GS) activity was relatively higher in UN and GN (Figure 4A), while glutamine-2-oxoglutarate aminotransferase (GOGAT) activity was remarkably reduced by AN and organic N (Figure 4B). In contrast, AN and organic N significantly increased the activity of NAD-dependent glutamate dehydrogenase (NAD-GDH). However, NAD-dependent GDH (NADH-GDH) activity was noticeably lower in AN and UN than in NN (Figure 4C,D). AN and organic N also increased glutamate decarboxylase (GAD) and GABA aminotransferase (GABA-T) activities with the exception of GABA-T in UN. (Figure 4E,F).

The expression of *NIA2.1* was substantially lower in AN and organic N in relation to NN (Figure S2A). Organic N sources also repressed the expression of *GS1*, *GS2*, *NADH-GOGAT*, and *Fd-GOGAT* (Figure S2B–E). The expression of *GDH* was downregulated by AN but upregulated by GN relative to NN (Figure S2F), while the expression of *GAD1* and *GABA-TP3* was remarkably upregulated by UN (Figure S2G,H). These results indicated that AN and organic N treatments attenuated plant N assimilation but enhanced GABA shunt, which could reduce the competition for C between N assimilation and secondary metabolism and promote C anaplerosis in the TCA cycle.

AN and organic N promoted the synthesis of aromatic amino acids (Try, Phe and Tyr) and aspartate family amino acids (Lys, Met, and Thr) but reduced glutamate and aspartate levels. However, glutamine was highly accumulated by AN and GN (Figure 2). Proline, arginine, and GABA were increased by organic N. In line with proline and arginine, spermidine was significantly accumulated in organic N as well.

**Figure 5:**
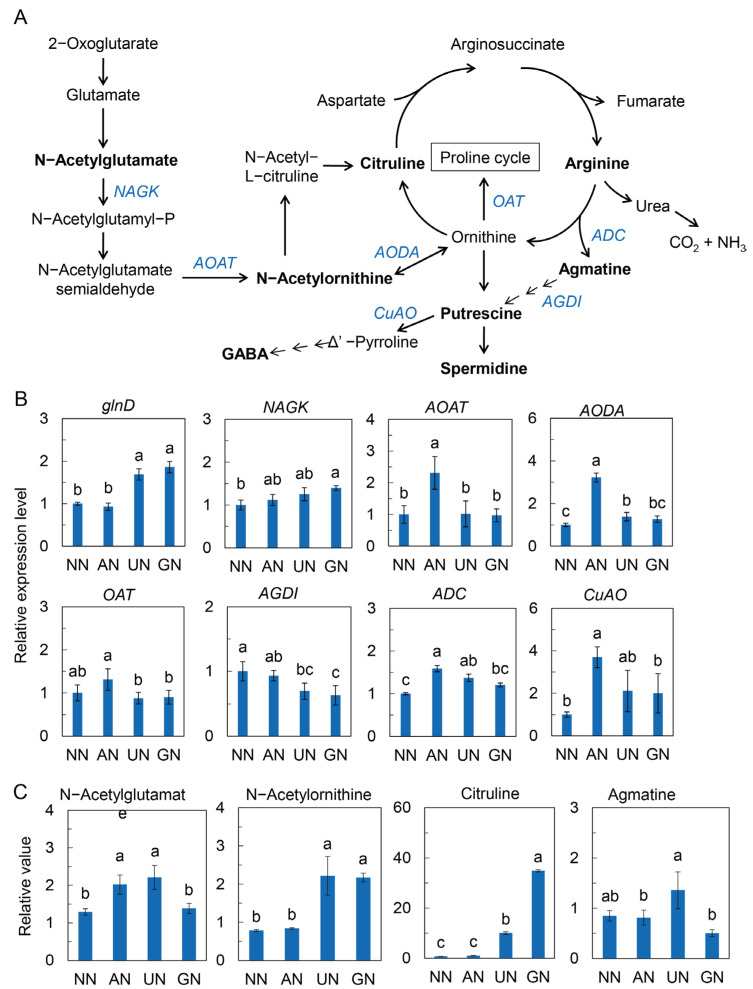
Arginine metabolism in response to N sources. (**A**) Arginine metabolic pathway. (**B**) Relative expression level of genes in arginine metabolism. (**C**) Relative levels of intermediate metabolites in arginine metabolism. The data were normalized to these in the NN treatment. Data were represented by means ± SD (*n* = 3). Different letters on bars indicate significant differences at *p* < 0.05 level according to one-way ANOVA combined with Duncan’s multiple range method. NN—nitrate nitrogen; AN—ammonium nitrogen; UN—urea nitrogen; GN—glycine nitrogen.

Further analyses of the arginine metabolism showed that AN and/or organic N upregulated the expression of *glnD*, *NAGK*, *AOAT*, and *AODA* and increased the levels of N-acetylglutamate, N-acetylornithine, and citrulline to a certain extent (Figure 5). Although *AGDI* expression was repressed by organic N, *ADC* and *CuAO* were significantly upregulated by AN, and, to some extent, by organic N. It was suggested that the arginine biosynthesis pathway and its downstream polyamine oxidation were strengthened by AN and organic N (Figure 5).

### 2.4. Phytohormone Levels Affected by N Sources

**Figure 6:**
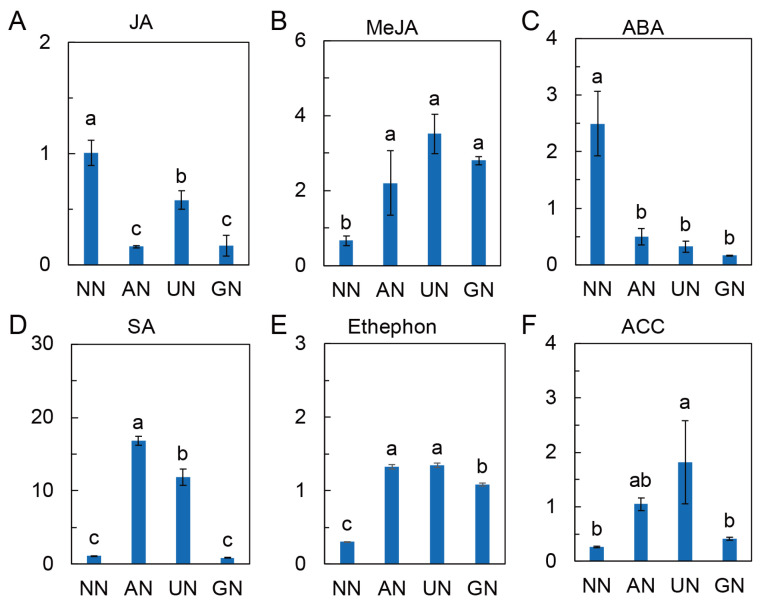
Phytohormone levels under different N source conditions. (**A**) Relative level of jasmonic acid. (**B**) Relative level of methyl jasmonate. (**C**) Relative level of abscisic acid. (**D**) Relative level of salicylic acid. (**E**) Relative level of ethephon. (**F**) Relative level of ACC. The data were normalized to NN and represented as mean ± SD (*n* = 3). Different letters on bars indicate significant differences at *p* < 0.05 according to one-way ANOVA and Duncan’s multiple range method. NN—nitrate nitrogen; AN—ammonium nitrogen; UN—urea nitrogen; GN—glycine nitrogen.

Jasmonate (including JA and MeJA), salicylic acid (SA), abscisic acid (ABA), 1-aminocyclopropanecarboxylic acid (ACC) and ethephon were detected in different N sources conditions. JA and ABA were remarkably reduced, while MeJA and ethephon were considerably increased by AN and organic N over NN (Figure 6A–C,E). SA and ACC levels were significantly higher in AN and UN compared to NN and GN (Figure 6D,F). The increases in SA and ACC were consistent with the increased precursors and intermediate metabolites and the expression of relative genes (Figures S3 and S4).

## 3. Discussion

### 3.1. N Sources Affect Diterpenoid Lactone Components Accumulation

N sources affect the components and content of bioactive ingredients in medicinal plants by modulating the expression of genes in the synthetic pathway [7,13]. Since the complete pathway for the synthesis of andrographolide has not yet been elucidated, studies for the genes of andrographolide biosynthesis mainly focus on those in the MVA and MEP pathways. Both pathways are involved in the biosynthesis of andrographolide in *A. paniculata* [35,36], but the dominant pathway is influenced by environmental factors [36,37]. It has been reported that NH_4_^+^ stimulated the MVA pathway in *Arabidopsis thaliana* [20]. Herein, we found that NH_4_^+^ mainly induced the expression of genes in the MEP pathway, while urea upregulated the genes in both the MEP and MVA pathways, and Gly mainly affected the genes in the MVA pathways (Figure 1B). It was suggested that the transcriptional regulatory mechanism of andrographolide biosynthesis varied with different N sources. Nevertheless, farnesyl pyrophosphate, the skeleton for the synthesis of geranylgeranyl pyrophosphate and subsequently diterpenoids, was extraordinarily highly accumulated under AN and organic N conditions, suggesting a similar metabolic regulatory mechanism of these N sources on andrographolide components.

In this study, we found that N sources affected the main components of diterpenoid lactones in *A. paniculata*. It is widely accepted that cytochrome P450 monooxygenases (CYP450s) are responsible for the biosynthesis of andrographolide and its derivatives after the formation of diterpene skeleton [38], although those genes have not yet been identified. Andrographolide, 14-deoxyandrographolide, neoandrographolide and dehydrographolide were generally increased under AN and organic N conditions, suggesting that *CYP450* genes are the key targets regulated by N forms in the biosynthesis pathway of andrographolide in *A. paniculata*. The results of this study could help us explore the *CYP450* genes in the synthesis pathways of andrographolide.

### 3.2. N Sources Reprogram C and N Metabolism

Photosynthesis is the initial C source for primary and secondary metabolism. Increasing photosynthesis facilitates plant secondary metabolite synthesis [39]. In this study, AN and UN promoted the biochemical processes of photosynthesis to a certain extent, as *V*_cmax_, *J*_max_ and *TPU* were higher than those in NN. Additionally, the photosynthetic rate at ambient CO_2_ concentration (~400 μmol·mol^−1^) was significantly higher in AN and UN treatments, suggesting that a greater assimilative productivity contributed to increased andrographolide biosynthesis in these N source conditions.

Apart from the production of assimilates, the partitioning of C metabolism also affects the relationship between primary and secondary metabolism [40]. Many studies showed that N-affected secondary metabolism was associated with partitioning C source between growth and defense [21,41], typically manifested as the alteration of the C/N ratio [7]. N sources affect the accumulation of sugars in plants [42]. Although the mechanism underlying the interconnection between sugar and secondary metabolite accumulation has not been well unveiled, there was a positive correlation between them in medicinal plants [10,21]. In the present study, the accumulation of sucrose and fructose was generally higher or not different from NN in AN, UN, and GN. The accumulation of sugars is the result of their anabolism and catabolism. The increased activities of SS and SPS and the reduced activity of PFK could be important factors in sugar accumulation. Gluconeogenesis also regulates the expression and activity of SPS, accelerating sucrose synthesis and accumulation [43]. We found that the activity of PEPCK, the rate-limiting enzyme of gluconeogenesis, was remarkably increased by AN, UN, and GN (Figure 3J). These results suggested that N-source-mediated sugar homeostasis could be involved in the regulation of andrographolide biosynthesis.

Nitrate reductase (NR) and PEPC play essential roles in coordinating the C and N metabolisms in plants [44]. In this study, the expression of *NIA2* was extremely suppressed by AN and organic N, and other N metabolic genes were also repressed (Figure S2), indicating that these N sources attenuated plant N assimilation activity. Our previous study proposed that high N assimilation activity is responsible for low andrographolide accumulation in *A. paniculata* [10,25]. N assimilation is the main process competing with secondary metabolism for C [41]. Thus, reducing N assimilation could stimulate the allocation of carbohydrates to secondary metabolism. The C skeletons required for N assimilation are replenished from glycolysis to the TCA cycle, the central metabolic hub for the C and N metabolism interactions [45,46]. An open mode of the TCA cycle promotes N assimilation by sustaining C skeleton availability [47]. Coleto et al. [20] reported that ammonium-enhanced anaplerotic routes are associated with the TCA cycle. PEPC in the anaplerotic pathway is believed to play a significant role in replenishing C for the TCA cycle via CO_2_ fixation to support N assimilation [48]. We observed that although the activity of PK was constant among N source treatments, both PEPC and PDH activities were reduced by AN and organic N. In addition, PEP and pyruvate contents were declined in these N source conditions. The above findings suggested that AN and organic N attenuated carbohydrate allocation from glycolysis to the TCA cycle and downstream N assimilation. As a result, more carbohydrates can serve as substrates for production of secondary metabolites and regulators such as plant hormones (discussed later).

### 3.3. The Center Metabolic Pathway in Mediating Reprogramming of C Metabolism

N assimilation requires 2-oxoglutarate (2-OG) for the C skeleton. 2-OG is derived from sugar respiration and amino acid transamination. In the former biochemical process, 2-OG is produced following glycolysis, the TCA cycle, and the anaplerotic pathway [49]. NAD-GDH oxidizes glutamate to produce 2-OG and NH_4_^+^. There is considerable evidence for a GDH shunt to return the C in amino acids back into C metabolism and the TCA cycle [50]. In the current study, AN-induced accumulation of 2-OG could be attributed to increased activities of ICDH and NAD-GDH and decreased activities of NADH-GDH (amination activity) and GOGAT. The accumulation of 2-OG, accompanied by reduced N assimilation, suggested that 2-OG was less likely to act only as a C skeleton. Indeed, 2-OG also acts as a cofactor for dioxygenase enzymes that are important in several phytohormone synthetic pathways and a signal for some enzymes sensing C and N status in plant cells [51]. It has been proposed that the accumulation of 2-OG controls C metabolic flux to N metabolism or other metabolic pathways [45,51]. Its accumulation indicates sufficient C supply for N assimilation, which could allow the allocation of more available C to fuel andrographolide synthesis.

It has been revealed that the P_II_ protein interacts with 2-OG, controlling C metabolic flux in N assimilation [51]. In the present study, we found that the expression of *glnD* (also known as uridylyltransferase and uridylyl removing (UR) enzyme, which converts P_II_ protein deuridylylation in N-rich conditions) was remarkably upregulated by UN and GN. Glutamine, the first organic product of N assimilation in plants, is commonly considered to be an important intracellular N status signal [52], of which accumulation indicates N enrichment in plants. It binds to the P_II_ protein, and it is only after binding that the P_II_ protein is able to form a complex with and activate NAGK and subsequently trigger the synthesis of arginine [53]. Arginine is an important N storage compound with the lowest C/N ratio in amino acids. It is critical for N reutilization in plants [54]. Here, we observed in AN and organic N treatments that glutamine level and the expression of *NAGK* and *glnD* were increased. Simultaneously, the expression of *AOAT* and *AODA* and/or the levels of intermediates of arginine metabolism such as N-acetylglutamate, N-acetylornithine and citruline were increased as well. It was indicated that AN and organic N sources enhanced arginine metabolism.

Degradation of polyamine derived from arginine metabolism is an important pathway for GABA synthesis in plants [55,56]. Here, we found that the expression of *ADC* and *CuAO* was upregulated by AN and organic N to some extent, which could impulse the synthesis of GABA. In addition, the increases in the levels of GABA and succinate, as well as the increments of GAD and GABA-T activities and their transcript levels, suggested that GABA shunt was strengthened by AN and organic N. Obviously, GABA shunt provides C and N to complement TCA cycle, which could regulate C flux derived from glycolysis. On the other hand, GABA can regulate cytosolic pH [57], which is considered an essential signal mediating plant secondary metabolism [16]. GAD and PEPCK were acidic inducible enzymes [15]; their activities were increased in plants exposed to ammonium [20]. They consume H^+^ via GABA synthesis and MDH-associated malate decarboxylation, respectively. The activities of GAD, PEPCK, and MDH were consistently elevated by AN and organic N. Our results suggested that GABA, accompanied by malate metabolism, not only regulated C in the TCA cycle but could also participate in plant secondary metabolism via regulating cytosolic acidification. However, the signaling mechanism of N-source-induced TCA metabolism alteration in the regulation of andrographolide biosynthesis is worthy of further study.

### 3.4. Plant Hormones Involve in N Source Regulated Andrographolide Synthesis

Phytohormones, including JA (and MeJA), SA, ABA, and ethylene, extensively participate in the signaling network of stimuli-triggered plant secondary metabolism [40,58]. The positive regulatory role of ABA in andrographolide biosynthesis has been reported by Guan et al. [59]. In the current study, however, the level of ABA in plants grown in AN and organic N was remarkably lower than that in NN, suggesting that ABA could weakly contribute to N sources-regulated biosynthesis of andrographolide compounds. MeJA and SA are defensive hormones that have profoundly influenced plant secondary metabolism in response to various environmental factors [33,60]. In this study, MeJA was significantly increased by AN and organic N, which aligns with the increases in andrographolide compounds, implying that MeJA could regulate andrographolide compounds biosynthesis under different N source conditions.

SA is another defensive hormone modulating plant secondary metabolism [60,61,62]. Shikimate and chorismate derived from PEP are important precursors for the synthesis of SA [63]. Ding et al. [64] reported that reduced N assimilation stimulates the synthesis of SA and increases plant immunity. We also observed that AN and organic N reduced N assimilation but increased SA synthesis, as revealed by increases in chorismate, phenylpyruvate, and phenylalanine, and decreases in shikimate and *trans*-cinnamic acid (Figure S3). The increased SA synthetic pathway implied that SA could also participate in biosynthesis of N sources-regulated andrographolide compounds in *A. paniculata*.

Ethylene and its precursor ACC had effective regulatory functions in plant secondary metabolism [65,66,67]. The N sources stimulate ethylene biosynthesis and signaling by upregulating the transcriptional levels of *ACS*, *ACO*, and *EIN3*, with a greater effect of ammonium relative to nitrate [68,69,70]. In this study, AN and organic N induced the synthesis of methionine, S-adenosyl methionine (SAM) and ACC, as well as the expression of *ACO* and *EIN3*. The level of ethephon, which is functionally similar to ethylene [71], was also increased by these N sources. It was suggested that ethylene could also be involved in the N-source-mediated biosynthesis of andrographolide compounds.

There is complex crosstalk between plant hormones in mediating plant secondary metabolism. For example, the interaction between ethylene and MeJA affects phenolic compounds in *Catharanthus roseus* [72], and the AP2/ERF family often acts as part of signaling cascades induced by JA controlling plant secondary metabolism [73]. Further studies are needed to reveal the specific roles of plant hormones in N sources-regulated andrographolide biosynthesis in *A. paniculata*.

## 4. Materials and Methods

### 4.1. Plant Materials and Growth Conditions

*Andrographis paniculata* of the Acanthaceae family was used as material in this study. The seeds of *A. paniculata* were obtained from the seed bank of the Guangxi Botanical Garden of Medicinal Plants and germinated on wet filter papers at room temperature (about 28 °C). Two weeks later, the germinated seedlings were transplanted to a seedling tray for continuous growth until three-pair leaf age in a mixed matrix containing vermiculite and perlite. The seedlings with two pairs of true leaves were fed with 1/10 strength of nutrient solution as described below. The N source was nitrate. Then, the seedlings were transplanted to pots filled with vermiculite and perlite (4:1) for N sources treatments. The seedlings were grown in a climatic room with a temperature of 28 °C, relative humidity of 60–70%, and a 14-h photoperiod of 200 μmol·m^−2^·s^−1^ photosynthetic active radiation (*PAR*).

Nitrate (NN), ammonium (AN), urea (UN), and glycine (GN) were offered by 2 mM KNO_3_ and 2 mM Ca(NO_3_)_2_, 6 mM NH_4_Cl, 3 mM CO(NH_2_)_2_, and 6 mM glycine, respectively, as the sole N source in each treatment. Other mineral nutrients in the nutrient solution contained 1 mM NaH_2_PO_4_, 2 mM KCl, 2 mM CaCl_2_, 1 mM MgSO_4_, 18 μM H_3_BO_3_, 0.15 μM ZnSO_4_, 0.15 μM CuSO_4_, 0.52 μM (NH_4_)_6_Mo_7_O_24_, 9.5 μM MnSO_4_, and 36 μM Fe-EDTA. In the NN treatment, KCl and CaCl_2_ were replaced by KNO_3_ and Ca(NO_3_)_2_, respectively, to provide NO_3_^−^ and maintain a balance of K^+^ and Ca^2+^ concentrations. The pH of the solution was adjusted to 5.8–6.0, and 10 mg·L^−1^ ampicillin was added to inhibit microbes. The nutrient solution was supplied twice a week at 200 mL per pot. A month later, plants grown in different N sources were sampled for analysis. NN was used as a control.

### 4.2. Photosynthesis and Leaf Nitrogen Allocation Measurements

The photosynthetic CO_2_ response curve (*A*-*C*_i_ curve) was measured using the LI-6400*XT* portable photosynthesis system (LI-COR, Lincoln, NE, USA). The fully expanded new leaves on the main stem were placed in the leaf chamber and adapted for 20 min at 1500 μmol·m^−2^·s^−1^ *PAR* and 450 ± 20 μmol·mol^−1^ ambient CO_2_ (*C*_a_). Then, the photosynthetic gas exchange was measured at a series of *C*_a_: 1500, 1200, 1000, 800, 600, 400, 300, 200, 150, 100, and 50 μmol·mol^−1^. The maximum carboxylation rate (*V*_cmax_), maximum electron transport rate (*J*_max_) and triose phosphate utilization (*TPU*) were calculated using the *A*-*C*_i_ curve according to the FvCB model [74] by a photosynthesis tool [75]. Photosynthetic N use efficiency (PNUE, μmol·g^−1^ N·s^−1^) was calculated as *P*_n_/SLN, where *P*_n_ (μmol·m^−2^·s^−1^) is the photosynthetic rate at 400 μmol·mol^−1^ CO_2_, and SLN was specific leaf N (g·m^−2^).

After photosynthesis measurement, the N and chlorophyll contents in leaves were determined based on leaf area. The total N was measured by digesting leaf samples in H_2_SO_4_-H_2_O_2_ and quantified by indophenol blue colorimetry method at 625 nm [76]. (NH_4_)_2_SO_4_ was used as the standard. The chlorophyll was extracted by immersing the leaf samples in acetone and alcohol (*v*:*v* = 1:1) for 24 h and measured spectrophotometrically at 663 nm and 645 nm [77].

### 4.3. Metabolites Analysis

Leaves were sampled and immediately frozen in liquid nitrogen and then stored at −80 °C for metabolite analysis. The frozen samples (about 100 mg) were ground into a fine powder with a mortar and pestle in liquid nitrogen. Then 1 mL cold extraction solvent (methanol/acetonitrile/H_2_O, 2:1:1, *v*/*v*/*v*) was added to the samples and adequately vortexed, followed by incubating the samples on ice for 20 min and centrifuging at 14,000× *g* and 4 °C for 20 min. The supernatant was collected and flowed through a 96-well protein precipitation plate, and then the elution was collected and dried in a vacuum centrifuge at 4 °C. For LC-MS analysis, the samples were re-dissolved in 100 μL acetonitrile/water (1:1, *v*/*v*) solvent and then transferred to LC vials.

For untargeted metabolomics of polar metabolites, the extracts were analyzed using a quadrupole time-of-flight mass spectrometer (Sciex TripleTOF 6600, Sciex, Framingham, MA, USA) coupled to hydrophilic interaction chromatography via electrospray ionization in Shanghai Applied Protein Technology Co.; Ltd. (Shanghai, China). LC separation took place on an ACQUIY UPLC BEH Amide column (2.1 mm × 100 mm, 1.7 µm particle size (Waters, Wexford, Ireland) using a gradient of solvent A (25 mM ammonium acetate and 25 mM ammonium hydroxide in water) and solvent B (acetonitrile). The gradient was 85% B for 1 min and linearly reduced to 65% in 11 min, and then reduced to 40% in 0.1 min and kept for 4 min, followed by an increase to 85% in 0.1 min. A 5 min re-equilibration period was employed. The flow rate was 0.4 mL·min^−1^, column temperature was 25 °C, autosampler temperature was 5 °C, and injection volume was 2 µL. The mass spectrometer was operated in both negative ion and positive ionization mode. The ESI source conditions were set as follows: Ion Source Gas1 (Gas1) as 60, Ion Source Gas2 (Gas2) as 60, curtain gas (CUR) as 30, source temperature 600 °C, and IonSpray Voltage Floating (ISVF) ± 5500 V. In MS acquisition, the instrument was set to acquire over the *m*/*z* range of 60–1000 Da, and the accumulation time for the TOF MS scan was set at 0.20 s/spectra. In auto MS/MS acquisition, the instrument was set to acquire over the *m*/*z* range 25–1000 Da, and the accumulation time for product ion scan was set at 0.05 s/spectra. The product ion scan was acquired using information-dependent acquisition (IDA) in a high-sensitivity mode. The parameters were set as follows: the collision energy (CE) was fixed at 35 V with ±15 eV; declustering potential (DP) at 60 V (+) and −60 V (−); exclude isotopes within 4 Da; and the candidate ions to monitor per cycle was 10.

### 4.4. Enzymes Assays

The frozen leaf samples (~0.1 g) were homogenized with 50 mM Tris-HCl buffer solution (pH 8.0, containing 2 mM MgSO_4_, 2 mM DTT and 0.4 M sucrose). The samples were centrifuged at 10,000× *g* and 4 °C for 10 min, and the supernatant was used for enzyme assays. The activities of glutamine synthase (GS), glutamate synthase (GOGAT), glutamate dehydrogenase (GDH), and isocitrate dehydrogenase (ICDH) were measured in a previous study [78].

The reaction medium of GS contained 40 mM hydroxylamine hydrochloride buffer (pH7.4, containing 40 mM MgSO_4_, 10 mM glutamate, 10 mM cysteine, and 1 mM EGTA), 10 mM ATP, and 0.5 mL enzyme extract. The total volume was 2 mL. After incubation of the mixture at 37 °C for 60 min, the reaction was terminated by adding acidic FeCl_3_ (0.37 M FeCl_3_ and 0.2 M TCA in 0.6 M HCl). The samples were centrifuged at 10,000 rpm for 10 min, and the absorbance at 540 nm (A_540_) was measured chromometrically. The blank was the absence of hydroxylamine hydrochloride. The GS activity was represented indirectly by the A_540_ per unit protein per hour.

The reaction mixture of GOGAT consisted of 10 mM a-ketoglutarate, 1 mM KCl, 37.5 mM of Tris-HCl buffer (pH 7.6), 0.6 mM of NADH, 8 mM of l-glutamine, and 0.1 mL of enzymes. The total volume was 1 mL. The activity was calculated by the average rate of NADH oxidation during the first 2 min after the start of the reaction.

The reaction medium of NADH-GDH contained 100 mM of Tris-HCl (pH8.0, containing 2 mM of α-ketoglutarate, 20 mM of NH_4_Cl), 2 mM of NADH, 10 mM of CaCl_2_, and 0.1 mL enzyme extract. The total volume was 1 mL. Activity was calculated by the average rate of NADH oxidation during the first 2 min after the start of the reaction.

The reaction medium of NAD-GDH contained 100 mM of Tris-HCl (pH 8.0, containing 100 mM l-glutamate), 10 mM of NAD^+^, 10 mM of CaCl_2_, and 0.1 mL of enzyme extract. The total volume was 1 mL. The activity was calculated by the average rate of NAD^+^ reduction during the first 2 min after the start of the reaction.

The reaction medium of ICDH contained 100 mM of K_2_HPO_4_-KH_2_PO_4_ (pH 7.5), 50 mM of MgCl_2_, 1 mM of NADP^+^, 30 mM of isocitrate, and 0.1 mL of enzyme extract. The total volume was 1 mL. The activity was calculated by the average rate of NADP^+^ reduction during the first 2 min after the start of the reaction.

Malate dehydrogenase (MDH) and NADP-malate enzyme (NADP-ME) activities were measured according to Kulichikhin et al. [79]. The reaction medium of MDH contained 100 mM HEPES-KOH (pH 7.5), 5 mM MgSO_4_, 0.2 mM NADH, 2 mM oxaloacetic acid (OAA), and 20 µL of enzyme extract. The total volume was 1 mL. The reaction was started by the addition of OAA. Activity was calculated by the average rate of NADH oxidation during the first 2 min after the start of the reaction. The reaction medium of NADP-ME contained 100 mM of Hepes-KOH (pH 7.5), 5 mM of MgSO_4_, 0.5 mM of NADP+, 5 mM of l(+)-malate, and 20 µL of enzyme extract. The total volume was 1 mL. The reaction was started by the addition of malic acid. The activity was calculated by the average rate of NADP^+^ reduction during the first 2 min after the start of the reaction.

Activities of glutamate decarboxylase (GAD) and GABA aminotransferase (GABA-T) were measured according to Deewatthanawong et al. [80]. GAD and GABA-T activities were represented as the generation rate of GABA and alanine, respectively. Frozen leaf samples (~0.1 g) were homogenized with 100 mM Tris-HCl (pH9.1, containing 10% glycerol, 1 mM DTT, 5 mM EDTA, 0.5 mM pyridoxal phosphate, and 1 mM PMSF). The samples were centrifuged at 10,000× *g* and 4°C for 10 min, and the supernatant was used for enzyme assays.

The reaction medium of GAD contained 100 mM potassium phosphate buffer (pH5.8), 40 μM pyridoxal phosphate, 3 mM l-glutamate, and 0.1 mL enzyme extract. After incubation at 30 °C for 60 min, 0.1 mL of 0.5 M perchloric acid was added to terminate the reaction. The produced GABA was measured in 1 mL reaction medium containing 200 μL potassium phosphate buffer (pH8.6), 150 μL 4 mM NADP^+^, 50 μL 2 U/mL GABAase, 50 μL 20 mM a-ketoglutarate, and 550 μL terminated reaction mixture. The activity was calculated by the average rate of NADP^+^ reduction during the first 2 min after the start of the reaction.

The reaction medium of GABA-T contained 50 mM of Tris-HCl (pH8.2, containing 10% glycerol, 1.5 mM of DTT, 0.75 mM of EDTA, 0.1 mM of pyridoxal phosphate, 16 mM of GABA, and 4 mM of pyruvate) and 0.2 mL of enzyme extract. After incubation at 30 °C for 60 min, 0.1 mL of 0.1 M sulfosalicylic acid was added to terminate the reaction. The produced alanine was measured in 1 mL reaction medium containing 50 mM of sodium carbonate buffer (pH10), 1.5 mM of NAD^+^, 0.02 U of alanine dehydrogenase, and 0.1 mL of terminated reaction mixture. The activity was calculated by the average rate of NAD^+^ reduction during the first 2 min after the start of the reaction.

Hexokinase (HK), fructokinase (FRK), glyceraldehyde-3-phosphate dehydrogenase (GAPDH), pyruvate kinase (PK) and phosphoenolpyruvate carboxylase (PEPC) activities were measured as previously reported [81].

The reaction system of HK contained 100 mM of Tris-HCl (pH 7.5), 0.2 mM of MgCl_2_, 2 mM of KCl, 1 mM of ATP, 1 mM of NAD^+^, 2 mM of glucose, 1 U of glucose-6-phosphate dehydrogenase (G6PDH), and 0.1 mL of enzyme extract. To measure the FRK activity, glucose was replaced by fructose, and 2 U glucose phosphate isomerase was added. The activity was calculated by the average rate of NAD^+^ reduction during the first 2 min after the start of the reaction.

The reaction medium of GAPDH contained 100 mM Tris-HCl (pH 8.4, containing 10 mM MgCl_2_ and 3 mM DTT), 5 mM ATP, 5 mM 3-PGA, 1 mM NADH, and 0.1 mL enzyme extract. The total volume was 1 mL. Activity was calculated by the average rate of NADH oxidation during the first 2 min after the start of the reaction.

The reaction medium of PEPC contained, in 1 mL, 100 mM of Hepes-KOH (pH 7.0), 100 mM of KCl, 10 mM of MgCl_2_, 0.2 mM of NADH, 1.5 mM of ADP and 24 units of l-malate dehydrogenase. The reaction was started by the addition of 10 mM PEP. Activity was calculated by the average rate of NADH oxidation during the first 2 min after the start of the reaction.

The reaction medium of PEPC contained, in 1 mL, 100 mM of Tris–HCl (pH 8.0), 10 mM of MgCl_2_, 25 mM of NaHCO_3_, 1 mM of dithiothreitol, 0.2 mM of NADH and 24 units of l-malate dehydrogenase. The measurement was started by the addition of 8 mM of PEP. Activity was calculated by the average rate of NADH oxidation during the first 2 min after the start of the reaction.

PEP carboxykinase (PEPCK) activity was measured according to Walker et al. [82] with some modifications. Crude enzyme extracts were obtained by homogenizing leaf samples in ice-cold 100 mM Tris-HCl (pH 9.8) containing 5 mm dithiothreitol (DTT). After centrifuging at 10,000× *g* and 4 °C for 10 min, the supernatant was used for determination of PEPCK activity in a continuous assay at 25 °C including 65 mM of Tris-HCl (pH 7.4), 100 mM of KCl, 0.3 mM of OAA, 1 mM of ATP, 10 mM of MnCl_2_, 4 mM of MgCl_2_, 71.5 mM mecaptoethanol, 0.1 mM of NADH, 2 units of pyruvate kinase, and 5 units of lactate dehydrogenase.

Phosphofructokinase (PFK) activity was determined by coupling the phosphorylation of fructose-6-phosphate with oxidation of NADH catalyzed by pyruvate kinase and lactate dehydrogenase using a PFK activity colorimetric assay kit (Acmec Biochemical Technology Co., Ltd., Shanghai, China). Citrate synthase (CS) and aconitase (ACOase) activities were measured using colorimetric assay kits (Michy Biology, Suzhou, China) according to the kit instructions.

Sucrose synthase (SS), sucrose phosphate synthase (SPS), and α-ketoglutarate dehydrogenase (α-KGDH) activities were determined using kits (Grace Biotechnology, Nanjing, China). Pyruvate dehydrogenase (PDH) was measured spectrophotometrically at 450 by monitoring the reduction of WST-8 using a kit (Michy Biology, Suzhou, China).

### 4.5. RNA Extraction, cDNA Synthesis, and q-PCR Profiling

Frozen leaf samples were pulverized in liquid nitrogen, and total RNA was extracted using a Trans Zol Up Plus RNA Kit (TransGen Biotech, Beijing, China). cDNAs were synthesized by the HiScriptIII RT SuperMix for qPCR (+gDNA wiper) Kit (Nanjing Vazyme Biotech Co.; Ltd.; Nanjing, China). The relative expression level of genes was analyzed by a ChamQ Universal SYBR qPCR Master Mix Kit (Nanjing Vazyme Biotech Co.; Ltd.; Nanjing, China) with a real-time qPCR system (QuantStudio 3, Thermofisher, Waltham, MA, USA). The primers used for q-PCR in this study are listed in Table S1. The sequences of genes were obtained from the genome of *A. paniculata* (NCBI, access number: ASM980555v1), and the primers were designed at primer BLAST of NCBI (https://www.ncbi.nlm.nih.gov/tools/primer-blast, accessed on 1 April 2023). The expression level of genes was normalized to the *Actin* gene (F: 5′-TAGAGAGTCCCCCGTATGCT-3′, R: 5′-CACAAGAATCCGACACGCAT-3′) by the method of 2^−ΔΔCT^ [83].

### 4.6. Statistical Analysis

The experiments were conducted using a completely randomized design. There were four biological replications in each treatment. The difference in physiological parameters among N source treatments was evaluated by one-way ANOVA combined with Duncan’s new complex range method. The significance was considered as *p* < 0.05. The heatmap of log_2_FC was performed at the Omicshare platform (https://www.omicshare.com/tools/home, accessed on 11 May 2023), and NN treatment was used as a control. A Student’s *t*-test was applied to determine the significance of differences between two groups of independent samples. *p* < 0.05 was used to evaluate significant changes in metabolites.

## 5. Conclusions

In this study, we revealed the physiological and molecular mechanism by which N sources reprogrammed C and N metabolisms to potentially regulate andrographolide biosynthesis in *A. paniculata*. In comparison to NN, the AN, UN, and GN increased the production of assimilates and reduced the partitioning of C metabolic flow to the TCA cycle and downstream N assimilation. Additionally, the arginine and GABA metabolisms were enhanced, which replenished C and N for the TCA cycle and, therefore, could reduce the C flow from glycolysis to the TCA cycle. As a result, the synthetic pathway of andrographolide can compete for more C, promoting the synthesis and accumulation of andrographolide and its derivatives. The reprogramming of the C and N metabolisms also resulted in the synthesis of salicylic acid and ethylene, which have positive regulatory effects on andrographolide biosynthesis.

## Data Availability

The original contributions presented in the study are included in the article/Supplementary Material; further inquiries can be directed to the corresponding author.

## Supplementary Materials

The following supporting information can be downloaded at: https://www.mdpi.com/article/10.3390/ijms25073990/s1.

## Abbreviations

α-KGDH, α-ketoglutarate dehydrogenase; ABA, abscisic acid; ACAT, acetyl-CoA C-acetyltransferase; ACC, 1-aminocyclopropane-1-carboxylic acid; ACO, ACC aoxidase; ACO, aconitase; ADC, arginine decarboxylase; AGDI, agmatine deiminase; AOAT, acetylornithine aminotransferase; AODA, acetylornithine deacetylase; CMK, 4-diphosphocytidyl-2-C-methyl-D-erythritol kinase; CPS, copalyl diphosphate synthase; CS, citrate synthase; CuAO, copper amine oxidases; DXR, 1-deoxy-D-xylulose-5-phosphate reductoisomerase; DXS, 1-deoxy-D-xylulose-5-phosphate synthase; EIN3, ethylene-insensitive3; FRK, fructokinase; FPP, farnesyl pyrophosphate; GABA, γ-aminobutyric acid; GABA-T, GABA aminotransferase; GAD, Glutamate decarboxylase; GDH, glutamate dehydrogenase; GGPPS, geranylgeranyl diphosphate synthase; GOGAT, glutamine-2-oxoglutarate aminotransferase; GS, glutamine synthase; HDR, 4-hydroxy-3-methylbut-2-en-1-yl diphosphate reductase; HDS, (E)-4-hydroxy-3-methylbut-2-enyl-diphosphate synthase; HMGR, hydroxymethylglutaryl-CoA reductase; HMGS, hydroxymethylglutaryl-CoA synthase; HXK, hexokinase; ICDH, isocitrate dehydrogenase; IDI, isopentenyl-diphosphate delta-isomerase; JA, jasmonic acid; *J*_max_, maximum electron transport rate; MCT, 2-C-methyl-D-erythritol 4-phosphate cytidylyltransferase; MDD, diphosphomevalonate decarboxylase; MDH, malate dehydrogenase; MDS, 2-C-methyl-D-erythritol 2,4-cyclodiphosphate synthase; ME, malate enzyme; MeJA, methyl jasmonate; MEP, 2-C-Methyl-D-erythritol 4-phosphate pathway; MK, mevalonate kinase; MVA, Mevalonate pathway; NAGK, N-acetylglucosamine kinase; OAT, ornithine aminotransferase; PDH, pyruvate dehydrogenase; PEP, phosphoenolpyruvate; PEPC, PEP carboxylase; PEPCK, PEP carboxykinase; PFK, phosphofructokinase; PK, pyruvate kinase; PMK, phosphomevalonate kinase; SA, salicylic acid; SS, sucrose synthase; SPS, sucrose phosphate synthase; TCA, tricarboxylic acid; *TPU*, Triose phosphate utilization; UDPG, uridine diphosphate glucose; *V*_cmax_, maximum carboxylation rate.
