# Supplementary material for: Nitrogen Sources Reprogram Carbon and Nitrogen Metabolism to Promote Andrographolide Biosynthesis in Andrographis paniculata (Burm.f.) Nees Seedlings
Source: Int J Mol Sci. 2024 Apr 3;25(7):3990. doi: 10.3390/ijms25073990 (PMC11012798; doi:10.3390/ijms25073990)
Supplement: Supplementary file 1 [file ijms-25-03990-s001.zip › ijms-2895469-supplementary.pdf]

Table S1 Primer sequences used for q-PCR in this study.

Genes	Forward (5'→3')	Reverse (5'→3')
Actin	TAGAGAGTCCCCCGTATGCT	CACAAGAATCCGACACGCAT
AACT	GATGTTGTTGTGGCAGGTGG	TCAGCACAATTCCCCATGCT
HMGS	GATCTTGAGAAGGCGAGCCA	CCGGCCAGAGAGCTACTTTT
HMGR1	GGAAAATCTCTCGCCGGACT	GAAGGAGTGGCCCTGCTATG
MK	ATGTTGTGGGAGCAGAGAGTG	AAGCATCATGGCCACCAACT
PMK	TCTGCTCCTGGGAAGGTTTTG	GGGTTCCTCGAGTCACTTCC
MDD2	GCCTGGTCTTCTCATTGGCT	CCCACTTGACGAATCCACCA
DXS	CAGGAAAACAGGCAAAGAGTA	TGGAGGAACGACGAGTAGAT
DXR	CGCCCAAAAAGGGGTTTGTT	TCTCTTGCCCGCACTGTATC
MCT	AAGACCTCTCGTGTTGGCTG	GCGAGTAGTAAGTCGTCGGG
CMK	ACTCCGGCTTTTGACGTTCT	AACTGAGGTGGATCGGGAGA
MDS	GGGCTTCCAGACATTGGTCA	CCCGAGCAACAGGCATAGAT
HDS	CCAAGGCTGCAGAATGAGGA	GAAACTCCGGGCAAGTGAGA
HDR	GCTTGTGGTAGGTGGGTTCA	TGCACCTGAAGTTATGCCGA
GGPS	AGGTGGTGGACGACATCCTA	CTCCCTCTTCAGCTGCTCTG
CPS	CCATGGGCGACGGAGAAATA	ACGCAGCTACGCATCCTAAT
UGT73AU1	GGGGGAGGTGAACTTGGATG	ACTCTTCCACCACGAGGGTA
NIA2	TTACCGGATCGGAGAGCTGA	CATCGTGTGAGAGCGAGGTT
GS1	AGTTTCAAGTCGGCCCTAGC	TTTGTGGCGAAGCGATAGGT
GS2	TCCACTCTCCACTCCTCACC	AGCCTCCGACCCAGATGTAT
NDAH-GOGAT	AGGAAGTCGAAGGTTCTGCG	AATCCCAGTTTCTCCGCCAG
Fd-GOGAT	CCCGGCGATCTCGTCAATTA	CCAAGGTCGATGTGCTGAGT
GDH	TGCTGATTTTAATGGCGCGG	TCCGGGTCAGTAGGATGGTT
GAD	TCGTGTCCAAGGATAACGGC	GTGTATGCGGGGACAATCCA
GABA-TP3	GGCGAGATTAGGGGAACAGG	ACTCGCACCAACATCCCATT
NAGK	GGACAAGGACAGGGCGATAA	CCGCGCCTCATAAACCAATC
ADC	GCCTCTCCCTACGCTTTCTC	AGTGAAGTAAGAAGCGCCCC

glnD	GCCGACGTAATGCGAACAAT	CCGATGGAACGACTGGAGTT
ACO	GCTGTCCAGTCGGAGATCAG	GTTGGAGACGGGGAGATGAC
EIN3	AGGGTCTTCAATCAGTGGCG	GCCCTTCTGGCTCACATTCT
AOAT	GACCGAGATGAACGGGCAAT	AGCGAAGTGTTGAGCGAGAA
AODA	CCTGGAGGTGGCATCAATCA	CTGGCGTTCTCCTCTGGAAG
OAT	ACTGCACTTACAGGCAACAC	AAAGTGGACTGGGACGTTCTG
AGDI	ACCGGAACCCCGATTTAACC	ACAACGCTCGTATTGTGGGT
CuAO	GTGGAGATGGTTGTGCCGTA	AGAAAGCACGATCGAGGCAA

Table S2 Parameters of *A-Ci* curve. V_{cmax}, J_{max} and *TPU* were calculated by the FvCB model [74]. Data were represented by means±SD (n=4). Different letters in the same column indicates significant difference at $P < 0.05$ level according to one-way ANOVA combined with Duncan's multiple range method. NN, nitrate nitrogen; AN, ammonium nitrogen; UN, urea nitrogen; GN, glycine nitrogen.

Treatment	V_{cmax} μmol·m^{-2}·s^{-1}	J_{max} μmol·m^{-2}·s^{-1}	*TPU* μmol·m^{-2}·s^{-1}
NN	47.82±0.35 ab	45.85±1.08 a	9.00±0.07 ab
AN	52.66±1.61 a	51.00±2.37 a	9.78±0.41 a
UN	50.09±2.04 ab	49.00±2.00 a	9.29±0.35 ab
GN	46.66±0.94 b	43.76±1.14 a	8.53±0.14 b

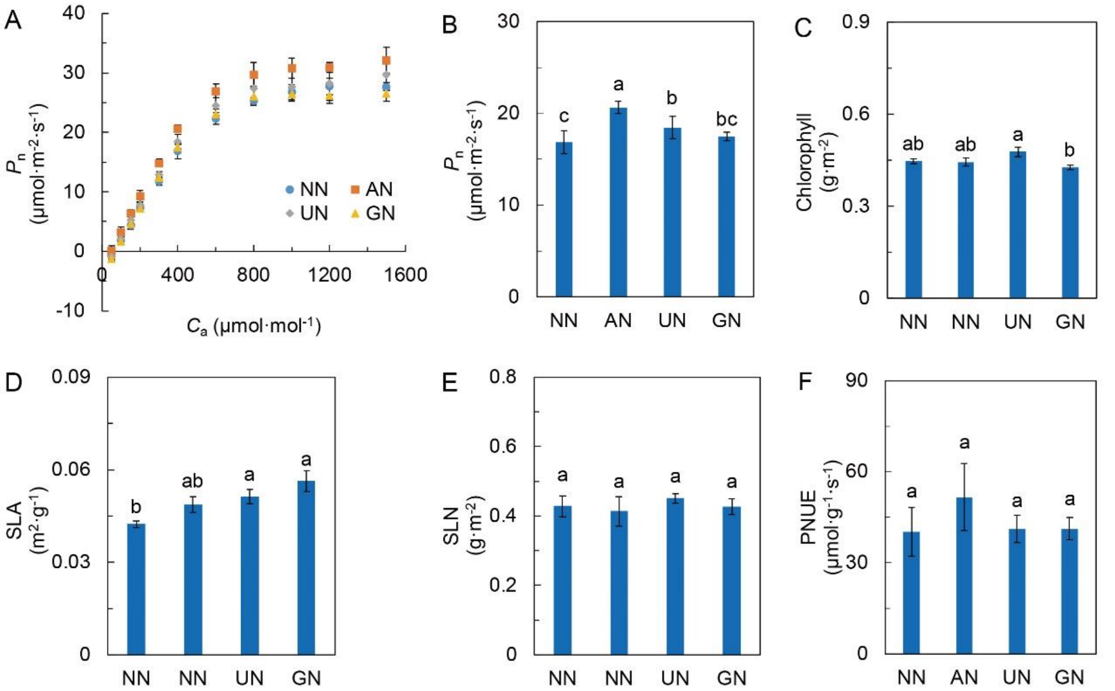

Fig.S1 Photosynthetic CO_2 response curve, chlorophyll, SLA and SLN in response to N forms in *A. paniculata*. **(A)** Photosynthetic *A*-*Ca* curve; **(B)** Photosynthetic rate at 400 μmol·mol^{-1} *Ca* (ambient CO_2 concentration); **(C)** Chlorophyll content based on leaf are; **(D)** Specific leaf area; **(E)** Specific leaf N content; **(F)** Photosynthetic N use efficiency. Data were represented by means±SD (n=4). Different letters in the same column indicates significant difference at $P < 0.05$ level according to one-way ANOVA combined with Duncan's multiple range method. NN, nitrate nitrogen; AN, ammonium nitrogen; UN, urea nitrogen; GN, glycine nitrogen.

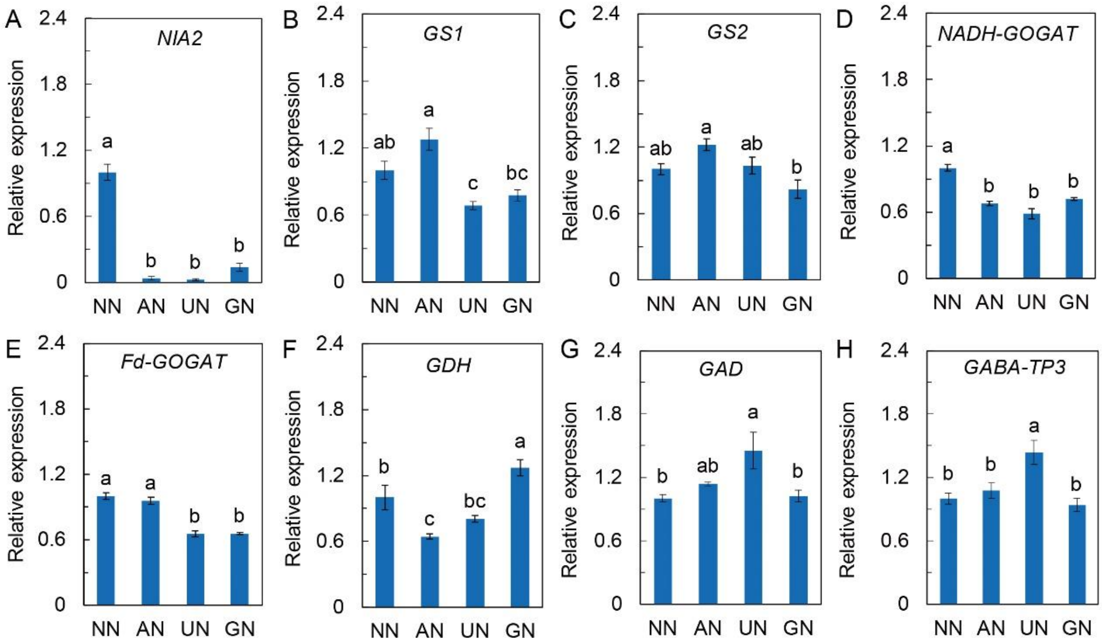

Fig.S2 Expression of N metabolic genes in response to N forms. Data were represented by means±SD (n=4). Different letters in the same column indicates significant difference at $P < 0.05$ level according to one-way ANOVA combined with Duncan's multiple range method. NN, nitrate nitrogen; AN, ammonium nitrogen; UN, urea nitrogen; GN, glycine nitrogen.

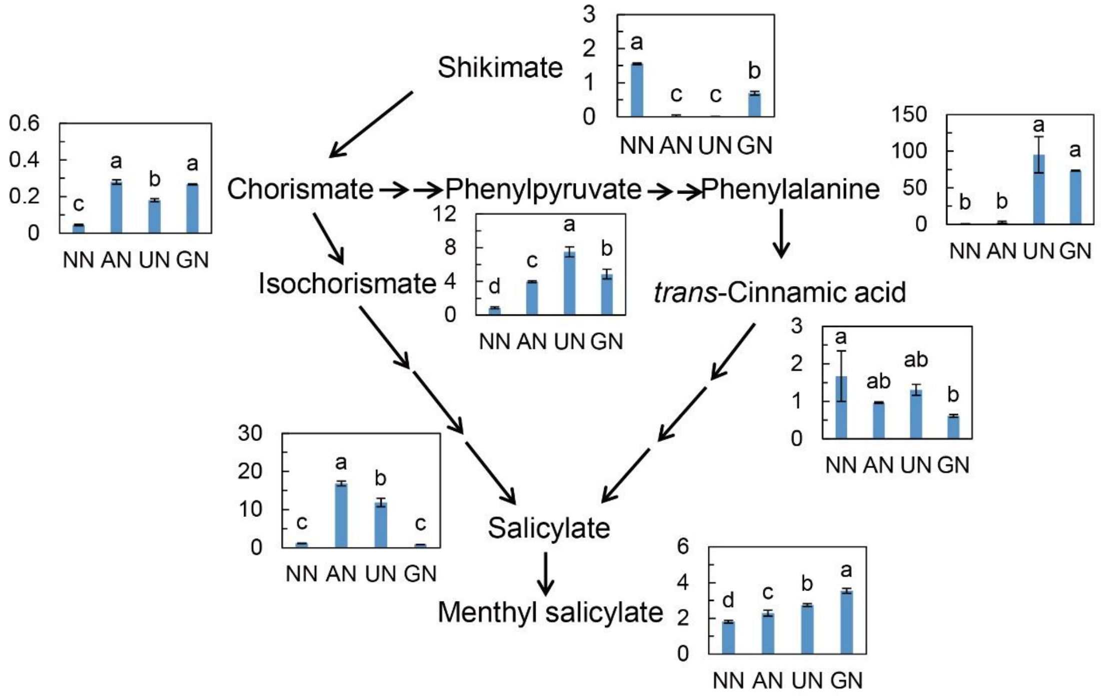

Fig.S3 Salicylic acid synthetic pathway in response to N forms. Data were represented by means±SD (*n*=4). Different letters in the same column indicates significant difference at $P < 0.05$ level according to one-way ANOVA combined with Duncan's multiple range method. NN, nitrate nitrogen; AN, ammonium nitrogen; UN, urea nitrogen; GN, glycine nitrogen.

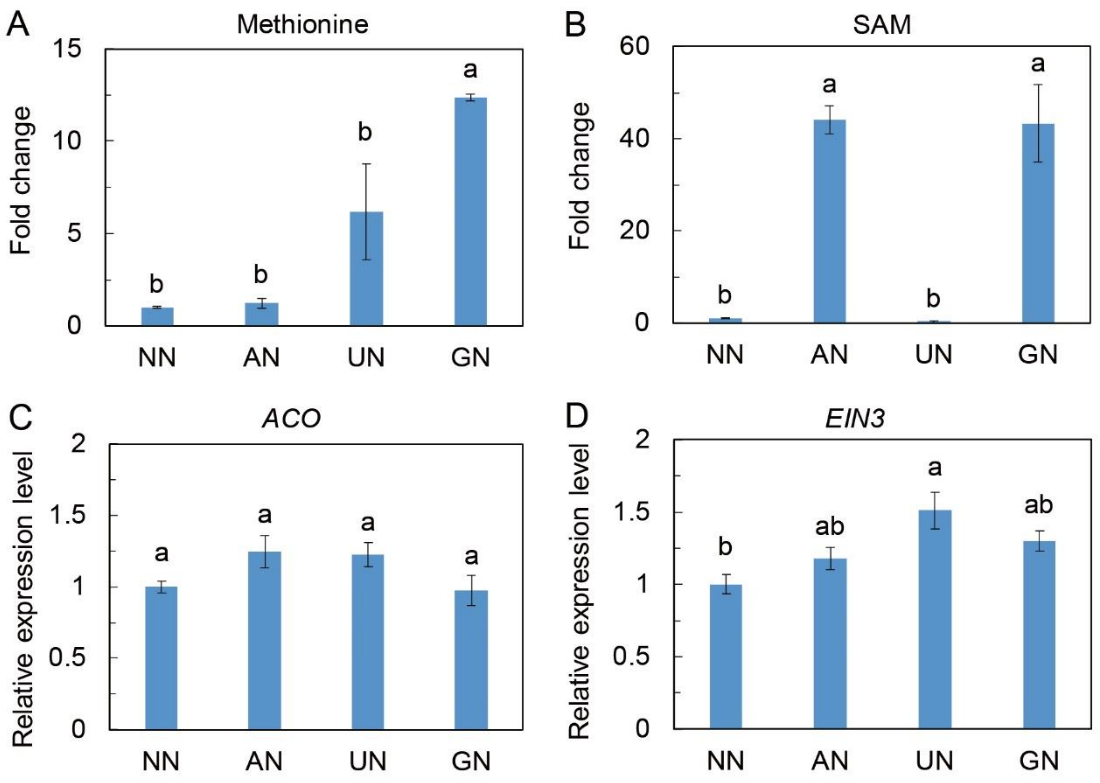

Fig.S4 Methionine and S- S-adenosylmethionine (SAM) levels and expression of *ACO* and *EIN3* in response to N forms. Data were represented by means±SE (n=4). Different letters in the same column indicates significant difference at $P < 0.05$ level according to one-way ANOVA combined with Duncan's new complex range method. NN, nitrate nitrogen; AN, ammonium nitrogen; UN, urea nitrogen; GN, glycine nitrogen.
